# Interventions to improve the quality of maternal care in Ethiopia: a scoping review

**DOI:** 10.3389/fgwh.2024.1289835

**Published:** 2024-04-17

**Authors:** Binyam Minuye Birhane, Yibeltal Assefa, Demeke Mesfin Belay, Gedefaye Nibret, Tigabu Munye Aytenew, Tewachew Muche Liyeh, Kelemu Abebe Gelaw, Yenework Mulu Tiruneh

**Affiliations:** ^1^ College of Health Sciences, Debre Tabor University, Debre Tabor, Ethiopia; ^2^ School of Public Health, University of Technology Sydney, Sydney, NSW, Australia; ^3^ School of Public Health, The University of Queensland, Brisbane, QLD, Australia; ^4^ Menzies School of Health Research, Charles Darwin University, Darwin, NT, Australia; ^5^ College of Health Sciences, Wolaita Sodo University, Wolaita, Ethiopia; ^6^ College of Health Sciences, University of Gondar, Gondar, Ethiopia

**Keywords:** Ethiopia, intervention, maternal, quality, scoping review

## Abstract

**Introduction:**

Quality improvement interventions have been part of the national agenda aimed at reducing maternal and neonatal morbidities and mortality. Despite different interventions, neonatal mortality and morbidity rates remain steady. This review aimed to map and synthesize the evidence of maternal and newborn quality improvement interventions in Ethiopia.

**Methods:**

A scoping review was reported based on the reporting items for systematic reviews and meta-analysis extensions for the scoping review checklist. Data extraction, collation, and organization were based on the Joanna Briggs Institute manual of the evidence synthesis framework for a scoping review. The maternal and neonatal care standards from the World Health Organization and the Donabedian quality of health framework were used to summarize the findings.

**Results:**

Nineteen articles were included in this scoping review. The review found that the studies were conducted across various regions of Ethiopia, with the majority published after 2013. The reviewed studies mainly focused on three maternal care quality interventions: mobile and electronic health (eHealth), quality improvement standards, and human resource mobilization. Moreover, the reviewed studies explored various approaches to quality improvement, such as providing training to healthcare workers, health extension workers, traditional birth attendants, the community health development army, and mothers and supplying resources needed for maternal and newborn care.

**Conclusion:**

In conclusion, quality improvement strategies encompass community involvement, health education, mHealth, data-driven approaches, and health system strengthening. Future research should focus on the impact of physical environment, culture, sustainability, cost-effectiveness, and long-term effects of interventions. Healthcare providers’ knowledge, skills, attitudes, satisfaction, and adherence to guidelines should also be considered.

## Introduction

Every day, pregnancy- and childbirth-related complications lead to the deaths of almost 800 women and 6,700 neonates globally. Moreover, approximately 5,400 stillbirths occur daily, with 40% of these fatalities occur during labor and delivery (1). Sub-Saharan African countries have recorded 546 maternal deaths per 100,000 live births, whereas developed regions have recorded only 12 deaths per 100,000 live births. Almost 94% of maternal deaths are associated with inadequate maternal care (2, 3). Ethiopia is a sub-Saharan African country that experiences a significant burden of neonatal mortality, with 30 deaths per 1,000 live births (4). It is crucial to prioritize quality maternal care to enhance the survival rates of both mothers and newborns (5).

Quality maternal and newborn care has been prioritized to catalyze action and support of the Sustainable Development Goal (SDG-3) global target of a maternal mortality ratio of less than 70 (5, 6). Almost half of the maternal population and more than 60% of neonatal deaths arise from poor quality care (7). Increasing access to healthcare services is needed to improve maternal and neonatal health (8); however, the paramount importance lies in quality of care.

The quality of care in the healthcare system is described from the perspective of healthcare providers, managers, and patients using elements such as safety, effectiveness, patient-centeredness, equity, and the provision of care (9). Improving the utilization of evidence-based guidelines through quality improvement initiatives proves effective, but implementing and maintaining them can be challenging (10).

Ensuring high-quality care is essential to reduce maternal morbidity and mortality. In 2016, the World Health Organization (WHO) established a quality of care (QoC) strategy for improving care for pregnant women and newborns. The strategy focuses on three areas of intervention to improve the quality of clinical care: enhancing the care experience and creating an enabling environment and system for quality care (11, 12), aligned with the strategies of ending preventable death and the Every Newborn Action Plan (13, 14). The quality of care could be affected by different factors, including a shortage of human resources (15); social, political, economic, and health system factors (16, 17); the knowledge, attitudes, and skills of healthcare providers; physical infrastructure; supply; leadership; provider's client relationship (18); mistreatment; and lack of support (19). Interventions were categorized based on the three systems of Donabedian's model of healthcare quality (input, process, and output) and the eight domains outlined in the WHO standard of care framework (20). The WHO recommends a comprehensive intervention strategy to make pregnancy safer: capacity development, increasing awareness, strengthening linkage, and improving the quality of services (21). In addition, interventions aimed at promoting health, such as enhancing the health of mothers and newborns, improving care provided at home, increasing community support for maternal health, increasing access to and use of skilled professionals, and empowering women, all work together to improve the quality of maternity care (22).

Evidence suggests that culturally appropriate maternity care interventions, such as home visits, formation of community-based health support groups, financial and community-based intervention packages, promoting awareness of women's rights, and educational training, improve the quality of maternal and newborn care (23–26). Moreover, improving the quality of midwifery education to meet international standards positively impacts the quality of maternity care (7). Interventions can be categorized as setting standards; implementing quality improvement programs; establishing performance-based initiatives (financial and non-financial); engaging and empowering clients; changing the clinical practices of healthcare workers; providing information and education to healthcare workers, managers, and policymakers; and implementing legislation and regulations for healthcare delivery (27).

In Ethiopia, the quality of maternity care became one of the areas of focus in 2014 and one of the four pillars of the Health Sector Transformation Plan (HSTP) II (2020–2025), which aimed at reducing maternal mortality to 279 per 100,000 live births, neonatal mortality to 21 per 1,000 live births, increasing skill delivery to 76%, and achieving antenatal care (ANC) coverage of 81% (28), which could be addressed through quality care interventions. Overall, quality improvement is observed throughout the continuum of care and improved emergency services (29, 30). Despite different maternal care quality improvement interventions in Ethiopia, maternal morbidity, adverse birth outcomes, and neonatal mortality rates remain steady. To the best of our knowledge, a scoping review targeting quality improvement interventions has not been conducted. As such, this review aims to identify, map, summarize, and inform priority research questions related to quality care interventions aimed at improving maternal and neonatal health in Ethiopia.

## Methods

### Identifying the research questions

The protocol was drafted based on the Preferred Reporting Items for Systematic Reviews and Meta-analysis Extension for Scoping Reviews (PRISMA-ScR) and has been registered on Figshare (31). A scoping review was used because of the broader nature (32) of quality maternal and neonatal care interventions. The review was based on Arksey and O’Malley's scoping review framework (33) and expanded to the Joanna Briggs Institute (JBI) framework for methodology consideration (34). We followed five essential steps of the Arksey and O'Malley methods in the review process. First, we identified the research questions. Second, we identified relevant studies. Third, we chose which studies to include. Fourth, we organized and recorded the data. Finally, the results were summarized and reported (35). The preferred reporting item checklist and explanation used for reporting are given in Supplementary File S1 (36).

BB and YA developed the research questions. The population, concept, and context frameworks were used to establish the eligibility of the research questions. The population included women/healthcare facilities, and the concept focused an intervention related to quality care within the Ethiopian context.

### Inclusion criteria

We included publications focusing on quality interventions (QI) to improve maternal and neonatal health in Ethiopia. All maternal and neonatal QI studies published in English were included. Papers with a study design, interrupted time series studies, before-and-after studies, program evaluations, randomized control trials (RCT), quasi-experimental designs, comparative cross-sectional studies, cohort studies, qualitative studies, and reports were included. Articles without full text or data that were challenging to extract were excluded.

### Search strategy

We thoroughly searched various bibliographic databases, including PubMed, Google Scholar, and the Cochrane Library, and performed manual search for unpublished sources to ensure comprehensive coverage of relevant literature. Medical subject headings, keywords, and free-text search terms were used. An extensive and comprehensive search was performed from the PubMed database using alternatives (all field options) (Supplementary File S2). A literature search was conducted between March 20 and June 4, 2022.

### Evidence screening and selection

First, a systematic search was conducted in all identified/accessed databases, search engines, and unpublished articles. Second, all retrieved studies were exported to Endnote version 7 (Thomson Reuters, London) reference manager, and duplicated studies were removed. Third, unrelated articles were excluded from the title review. Two investigators (BB and DB) independently screened titles, abstracts, and full texts to determine the eligibility of each study. During the review process, articles lacking full text were identified through discussions with the reviewers. Disagreements were resolved by consensus or a third party (YT).

### Data extraction

The data extraction tool was developed using the JBI manual of the evidence synthesis framework for scoping reviews (37). The extracted data were based on several tools, including author(s)/year of publication, study setting, aim/purpose, methods employed, type of intervention/comparator (duration of intervention), and key findings (Table 1).

**Table 1:** Characteristics of included studies.

Author (s) and year	Study settings	Objective	Study design	Intervention(s) type	Methods	Key findings
Nigussie et al. (38)	Amhara, SNNP, Oromia, and Tigray	- To improve delivery, timeliness and coverage, quality, and referral of RMNCH services - To bridge the communication gap between HCW and HEW using mHealth	Process evaluation	Mobile and electronic health (mHealth)	• Provision of mobile for pregnant women, HEWs, and HCPs • Providing training on the application • Performance monitoring and trace defaulter • mHealth applications-based supportive supervision • Registration and prioritization of maternity care services (ANC, delivery, and PNC services) • Providing automated job aids for HEWs • Referral and follow-up • mHealth information exchange between the health posts and health centers • Client's notification of appointment reminders using SMS	• Improve real-time communication b/n healthcare providers. • Improve timely identification and registration of pregnant women. • Adherence to treatment protocols • Provides reliable, quality, and on-time data for action • Supports access to clients' previous and current clinical information • Provides dynamic job aids to improve clinical skills and client counseling
Hagaman et al. (12)	Amhara, SNNP, Oromia, and Tigray	To evaluate the impact of QI health systems’ intervention on MCH outcome (feasibility of complex, low-cost, health-worker-driven improvement Interventions)	Quasi-experimental, interrupted time series approach	Scaling QI health systems’ intervention [from September 2016 to September 2018 (32 months)]	• QI initiative • Formation of quality improvement teams • QITs attended four structured learning sessions (provide training on QI, experience sharing, peer learning, and intensive coaching followed by the implementation of team-initiated QI change ideas and troubleshooting • Data extraction from facility paper registers and validation	• Improved HCW's adherence to a safe child practice • Increased attending at least four ANC visits from 64.1% to 75.3% • Increased rate of syphilis testing increased from 54.7% to 68.5% • Increase PNC visit within 48 h of discharge from 49.4% to 58.2% • Increase newborn-appropriate treatment in neonatal sepsis, kangaroo mother care, and birth asphyxia • No impact on skilled delivery
Ayalew et al. (39)	Amhara, SNNP, Oromia, and Tigray	To see the effect of Standard-Based Management and Recognition (SBM-R) on MNH providers' performance	Post-only intervention and comparison evaluation design	Standard-based management and recognition (SBM-R) quality improvement intervention (from March 2011 to June 2014)	• Introduce the SBM-R approach • Develop 10 technical areas and standards (80 standards for hospitals and 79 for health centers) • 3 round training on SBM-R for providers and managers • Training on (BEmONC) and regular follow-up • Baseline assessment for gap identification • Provide essential equipment and supplies for MCH service • Establish quality improvement team • Regular follow-up (every 6 month) using direct structured observations, document reviews, and provider interviews • The ANC portion of the tool consisted of 53 tasks in 8 skill areas; the labor and delivery section included 105 tasks in 10 skill areas; and the PNC portion included 9 tasks in 2 skill areas	• Almost no difference in ANC performance (63.4% vs. 61.0%) • Average performance score for uncomplicated labor and delivery was higher among the intervention groups (77.5% vs. 65.6%; *p* = 0.002) • Average performance score for immediate PNC service was higher among the intervention groups (72.8% vs. 50.6%; *p* = 0.001) • Average performance score was higher among the intervention groups in rapid initial assessment (60.6% vs. 42.8%, p = 0.019), care during labor (81.1% vs. 66.0%, *p* = 0.001), and immediate newborn care (76.9% vs. 61.9%, *p* = 0.013)
Biadgo et al. (40)	SNNP, Amhara, Oromia, and Tigray	To assess the quality of maternal and neonatal healthcare provision using the national MCH quality care standards and strengthen and develop a sustainable, self-sufficient healthcare system	Facility-based cross-sectional	Institute for healthcare improvement project using a district-wide collaborative approach	• Develop quality standards (28 items for input, 13 items for process, and 4 items for outcome) related to health-seeking behavior • Establish quality healthcare collaborative demonstration and learning sites • Identify gaps that affect performance • Interview • Observation • Document review of patient cards	• The mean quality score of input (infrastructure, availability of equipment/supply, and essential drugs) was 62% • The quality of the process component was 43% • 53% of mothers were assessed for danger signs at admission • Out of 1,920 cases, only 38% of newborns were given vitamin K and a mere 35% had skin-to-skin contact with their mothers and breastfed within 1 h of delivery • The quality of the maternal and neonatal health output component was 48% • 70% of births were attended by skilled health personnel • The mean score for overall complication management was 38% • It was found that only 11% of postpartum hemorrhage cases were handled according to the protocol, and a significant 63% of females with pre-eclampsia received IV/IM MgSO_4_ treatment • According to the established benchmark for quality of care in maternal and newborn health, a mere 15.6%, 9.3%, and 10.7% of healthcare facilities have successfully met the required standards for input, process, and output quality, respectively • Hospitals and health centers achieved 79% and 59% of the input standards, 58% and 41% of the process standards, and 62% and 46% of the output standards, respectively
Gebrehiwot and Tewolde (41)	SNNP, Amhara, Oromia, and Addis Ababa	To initiate a facility-based review of maternal deaths and near misses	An in-depth review	Ethiopian Society of Obstetricians and Gynecologists (ESOG) project to review maternal death (integrating the MDR and NMR processes) (November 1, 2010 and February 1, 2011)	• Establish a technical review • Establish a standing committee • Conduct a maternal death and near-miss review	• A total of 35,047 deliveries, which included 7,181 cesarean deliveries, 32,541 live births, and 2,604 stillbirths • A total of 2,774 cases were reviewed; 2,568 were near misses and 206 were maternal deaths • The maternal mortality ratio (MMR) in the facilities was 728 per 100,000 live births. In addition, the near-miss rate was 9,079 per 100,000 live births, and the case fatality rate was 8% • 76% of maternal deaths were attributed to direct obstetric causes, and 7% were due to indirect obstetric (4.8% were due to anemia) • 87.6% of women were critically ill, and 4.4% women died on arrival; 70.2% of women were delayed at home, 48.1% were delayed due to lack of transportation to reach the nearest appropriate health facility, and 34.7% were delayed in receiving care owing to a shortage of skilled health professionals or a lack of appropriate medical supplies • 23.6% of women who sought help and medical attention had no access to appropriate health facilities • 55.9% of women did not receive prenatal care • Date of delivery was registered for 1,434 (51.7%) women, while date of discharge was recorded for 1,356 (48.9%) • The ratio of live births to stillbirths among the delivered cases (*n* = 2,124) was 1:2 • The partograph was used for only 219 (39.9%) eligible cases (*n* = 549)
Kassa and Mokgadi (42)	North Wollo, Amhara Region	To assess the effectiveness of the mHealth intervention in MCN quality care (improve communication between HCWs)	Pre–post intervention	mHealth for 24 months	• Web application for registration and SMS engine for HCWs • Visiting pregnant women at Health Center marked with unique identification number. • The system generates four SMS reminders in connection with ANC visit based on calculation of GA (at GA of 26, 32,36, and 39 weeks); one SMS at 30, 14, 6, and 2 weeks; at day 1, day 3, and day 7 of PNC; three schedules at 6th, 10th, and 14th weeks for Penta vaccine	• Improved in 4 and more ANC visits (13.8% at baseline to 64% after mHealth intervention) • Timeliness to start (44.5% and 77.3%) • Institutional delivery (35.0% and 71.2% • PNC within 6 h of birth (23.8% at baseline and 84%) • Penta-3 vaccination coverage (61.5% and 70.4%)
Dadi et al.(43)	In 9 regions of Ethiopia	To estimate the effect of place of ANC-1 visit and adherence to MOH's ANC visit recommendations, institutional delivery, and PNC	National HEP assessment survey (secondary data)	Health extension program	Data collected during the health extension program	• The place of ANC-1 visit does not have a significant effect on the completion of the continuum of care • The mean availability score of medical equipment at the health post was 7.98, and 1.44% of the HPs do not have any medical equipment • Among women who have attended at least ANC-1 visit, 14.8% completed CoC • More than half (55.5%) of the women were not told at least one danger sign of their previous pregnancy • Two-thirds (64%) of women delivered their second youngest child at home • 92% took at least one ANC visit, but only 25% took PNC • Only 13.88% of the cohort completed the continuum of care, and 6.6% of them received MOH-recommended ANC • Adherence to ANC visits to the MOH recommendations improves the continuum of care
Getachew et al. (44)	In 9 regions of Ethiopia	To assess the care received by mothers and newborns during antenatal and delivery care	Institution-based survey	Standard care	• Interview and observation	• About 29% of women received the full components of AMTSL • Low knowledge of PPH management • Magnesium sulfate was rarely available in labor and delivery wards (only available in 3 out of 19 facilities • Partograph use was very low • Knowledge of the signs of obstructed labor was low • Diagnoses and management of asphyxia were low • Providers asked the client about at least one danger sign in only 34% of the cases observed • Providers asked the client about at least one complication during previous pregnancies in 27% of cases • Only half of the women in labor were greeted respectfully by the provider, and only 13% of women were asked if they had any questions • 66% of women were supported by the provider during labor in a friendly manner • Explanations of procedures and what would happen during labor were offered in about 35% of the laborers observed • Only 44% of the women were draped to protect their privacy • Only 12% of newborns were placed skin-to-skin • Only 18% of newborns received all elements of essential newborn care • There was only 48% adherence to thermal control
Lund et al. (45)	Oromia region	To assess the effects of the safe delivery app (SDA) on perinatal survival and healthcare workers' knowledge and skills in neonatal resuscitation	Randomized control trial	SDA mHealth training	• Mobile phone intervention with safe delivery application (SDA) • Standard care • Training on basic emergency obstetric and neonatal care • Data collection for secondary outcomes at 6 and 12 months • Follow women from delivery to 7 months for perinatal outcome	• SDA was an effective method to improve and sustain the healthcare workers' knowledge and skills in neonatal resuscitation • Perinatal mortality was not significantly reduced after the intervention
Sibley et al. (46)	Amhara and Oromia	To improve the completeness of maternal and newborn healthcare provided by the team of HEWs, community health development agents, and TBAs	Project evaluation (pre–post intervention)	Maternal Health in Ethiopia Partnership (MaNHEP) project (3.5 years project)	• Community-based maternal and newborn health training program, continuous quality improvement, behavior change communications • Monthly quality improvement monitoring	• Improvements in the completeness of maternal and newborn healthcare provision • Improved providers' confidence and skills in birth care • Improved identification of pregnant women, enrollment of pregnant women in ANC • Improved perinatal outcomes
Desta et al. (47)	Amhara and Oromia	To see the effect of the mobile video show on community knowledge, attitudes, and beliefs toward MCH service utilization	Project evaluation (qualitative and quantitative)	MaNHEP project	• Use mobile video shows for behavioral change on MCH	• Mobile video shows promote access to behavior change communication on MCH, bringing about desired changes in knowledge and beliefs • Improve recalling of maternal complications • Retain key messages
Asefa et al. (48)	SNNPR, Tigray, and Oromia	To see the effectiveness of respectful maternity care (RMC) interventions	Interventional mixed methods design (pre–post-quantitative and post-intervention qualitative)	Respectful maternity care project	• Training manual development and RMC videos • 3-day training on respectful maternity care • Consultative meetings with managers • Coaching	• Prior to the intervention, it was reported that 39.1% of participants witnessed examinations without privacy, and 21.9% reported the use of physical force. In addition, 29.7% of women admitted that they were mistreated • The awareness of women's rights during childbirth and their perceptions and attitudes toward RMC have been improved among providers • Positive perception on 8 RMC domains increased from 21.9% to 35.9% • Belief on not necessary to seek verbal consent from a woman prior to conducting a vaginal examination (15.6% pretest vs. 10.9% during post-test) • It was believed that nurses and doctors were unable to alter the procedures in the delivery room, and this perception worsened from 17.2% in the pretest to 18.7% in the post-test • The video helped providers to see their care from their clients' perspectives • The training helped in finding potential root causes of mistreatment and developing solutions
Mengistu et al. (49)						
Mihret et al. (50)	Amhara	To reduce disrespectful and abusive maternal care	Pre–post intervention mixed methods	RMC project	• Route cause analysis at baseline • Provision of 5-day training on RMC • Prepare standard written guidelines and protocols on RMC • Waiting room construction • Improving infrastructure such as availing screening or curtains, equipment, essential drugs, and supplies • Supportive supervision and mentoring, and staff motivation	• Initially, there are inadequate monitoring and evaluation systems, insufficient knowledge, and skills among staff regarding respectful care, and low provider motivation. In addition, there is missing medical equipment, such as ultrasound and blood pressure apparatus, delivery coaches, and crucial drug supplies needed for maternal health services, and the working environment is poor • In addition, there is a lack of written policies detailing the responsibilities of healthcare providers in the RMC process, as well as a professional code of conduct and ethics for providers working in labor wards and ANC clinics. Furthermore, there is an inadequate system in place for reporting illegal, incompetent, or impaired practices • Disrespect and abuse during pregnancy and childbirth decreased by 55.9% (reducing from 71.8% to 15.9%) • Physical abuse during maternal care was reduced from 61% to 15.4% • Non-confidentiality care, discrimination care, and abandonment or denial of care reduced from the baseline by 54.8%, 59.3%, and 68.4%, respectively • Non-consented care domain decreased by 54.9%
Berhanu et al. (51)	SNNP, Amhara, Oromia, and Tigray	To see the effect of CBNC on MCH services	Program evaluation (pre–post survey)	Community-Based Newborn Care (CBNC) program	• Training of HEWs on 9 components of CBNC • Reporting high utilization for integrated CBNC • Having a strong linkage within their primary healthcare units • Having a well-established health extension program • Having functional Women's Development Army networks	• The percentage of women who had at least one ANC visit increased by 15%, and those who had four or more visits increased by 17% (from 36% to 53%), and there was a 40% increase in the promotion of institutional delivery (from 22% to 62%) • Among women with at least one ANC visit, the proportion who reported giving a urine sample increased by 18%, receiving a syphilis test by 8%, and receiving iron folate by 9% • There has been a decline in the percentage of women who reported receiving guidance on birth preparedness by 7%, nutrition advice by 10%, and HIV testing by 19% • The percentage of newborns receiving PNC visits within 48 h of birth has decreased. There has been a 6% decrease for home deliveries and a 14% decrease for institutional deliveries • Skin-to-skin contact increased by 11% • Delay in newborn's bathing increased by 14% for home delivery
Villadsen et al. (52)	Oromia	To improve maternity care by ANC strengthening	Pre- and postintervention (ANC intervention)		• Supply equipment needed for ANC • Training of health staff and laboratory staff • Development of health education materials • Seminar with TBAs • Adaption of guidelines • Supervision	• Improved health education on danger signs during pregnancy • Improved laboratory testing (urine test and blood tests other than HIV) • Improved health problem identification • Increased in the proportion of women waiting less than 1 h • Improved women's satisfaction with the service • No effect on the frequency of physical examination performed and conduct of health professionals
Tesfaye et al. (53)	Amhara and Oromia	To promote community maternal and newborn health (CMNH) family meetings and labor and birth notification to improve PNC	Baseline and end-line cross-sectional survey	A community-based, collaborative quality improvement approach (MaNHEP) project	• Training HEWs, community health development agents, and traditional birth attendants (TBAs) in maternal care • Training for pregnant women and their family care giver in their 2nd and 3rd trimesters in maternal and newborn care including PNC through community maternal and newborn health (CMNH) family meetings • Collaborative quality improvement focusing on promotion of pregnancy identification, antenatal care registration, CMNH family meeting attendance, labor and birth notification and PNC within 48 h of birth by a HEWs • Behavior change communications	• The percentage of newborns receiving a PNC from skilled providers or HEWs within 48 h of birth significantly increased in Amhara (from 5% to 51%) and Oromiya (from 15% to 47%) • Women who received any ANC visit from a skilled provider or HEWs were more likely to receive PNC • Women who participated in two or more CMNH family meetings alongside their family members had a significantly higher chance of receiving PNC within 48 h from a skilled provider than those who attended less than two meetings. Women whose most recent birth was attended by HEWs/HCWs received PNC
Lindtjørn et al (54)	SNNPR	To determine the effects of several coordinated interventions (BEmOC and CEmOC) on effective coverage and reduce maternal deaths	Pre–post intervention	Healthcare system strengthening interventions	• Equipping institutions with trained staff on BEmOC and CEmOC provides essential and basic equipment • Regular monitoring and supervision • Community-based birth registration system	• During the intervention period, there was a significant decrease in MMR, with a decline of 64% from 477 to 219 deaths per 100,000 live births. The reduction in MMR was particularly pronounced in the woreda, where CEmOC functions • Four or more antenatal controls increased by 20% • There has been an improvement in the number of women referred for delivery services. The percentage of women delivering at home decreased by 20.4%, from 89.8% to 69.2% • Decline in the use of traditional birth attendants • The number of women referred to hospitals increased by 3.3% and to health centers by 7.2% more in CEmOC areas • Stillbirths reduced by 46% (from 14.5 to 7.8 per 1,000 births) • Those having road access to health facilities and those residing near health facilities had a lower mortality risk
Bitewulign et al. (55)	Amhara, Oromia, Tigray, and SNNP	to evaluate the effect of integrating the use of the World Health Organization Safe Childbirth Checklist (WHO-SCC) into a district-wide system improvement collaborative program designed to improve and sustain the delivery of essential birth care practice	Time series study		• Integrating the use of the World Health Organization Safe Childbirth Checklist (WHO-SCC) • Training on the checklist • Three “clinical bundles” were created from the WHO-SCC: on admission, before pushing, and soon after birth • Assess adherence monthly by reviewing charts of live births • Coaching • Observation • Document review • Triangulation of the checklist with the document review	• Improved adherence and quality of labor and delivery

### Collating, summarizing, and reporting the results

The scoping review findings were reported following the PRISMA-ScR guidelines. Data were presented using text, figures, and tables to describe the concept, population, and context. The interventions were classified into three systems based on Donabedian's model of healthcare quality (input, process, and output) and eight domains of the WHO's standard of care (56).

## Results

### Search results

**Figure 1:**
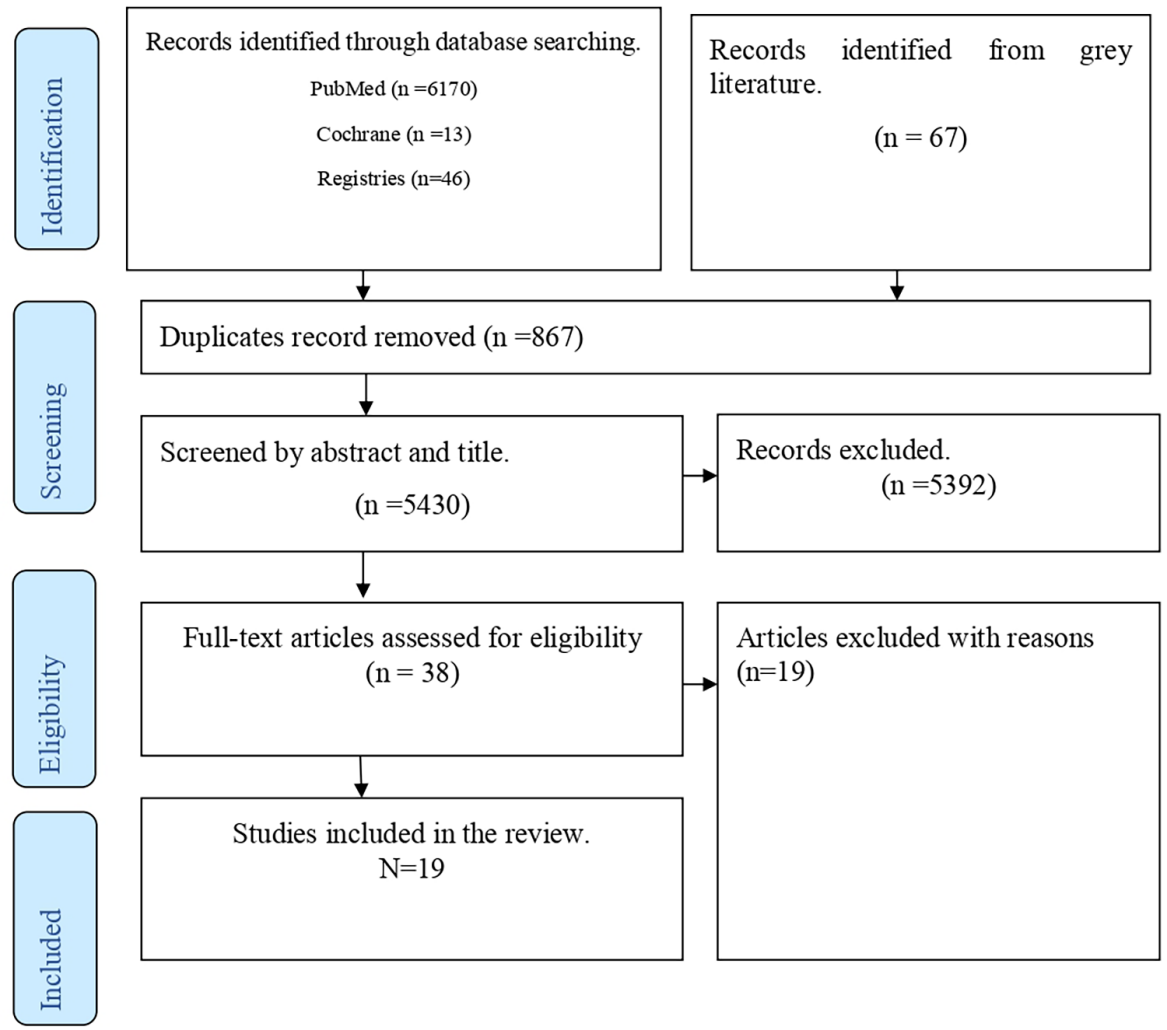
PRISMA flow diagram.

Articles were searched from PubMed (*n* = 6,170), Cochrane Library (*n* = 13), Registries (*n* = 46), and gray literature (*n* = 67), yielding a total of 6,296 articles, of which 5,430 remained after duplication removal. Following title and abstract screening, 38 articles were reviewed for full text. Finally, 19 articles were selected for inclusion in the scoping review (Figure 1).

### Characteristics of included studies

Nineteen studies were included in the review. As per the review, *n* = 2 studies were conducted in all regions of Ethiopia; *n* = 6 studies were conducted in four regions of Amhara, Oromia, SNNP, and Tigray; *n* = 1 study was conducted in three regions of Amhara, Oromia, and SNNP; *n* = 2 studies were conducted in three regions of SNNP, Tigray, and Oromia; *n* = 3 studies were conducted in two regions of Amhara and Oromia; *n* = 2 studies were conducted in Oromia; *n* = 2 studies were conducted in Amhara; and *n* = 1 study was conducted in SNNPR. Almost all articles were published after 2013, and only one study was conducted in 2011. As per the current review, nine studies (12, 38–42, 46, 47) focused on maternal and child health (MCH) [ANC, intrapartum, postnatal care (PNC)] intervention, two studies (43, 49) focused on ANC intervention, three studies (41, 44, 45) focused on intrapartum, two studies (51, 52) focused on PNC, and three studies (48–50) focused on respectful maternity care.

Most studies (*n* = 10) were pre–post intervention (project evaluation) studies (42, 46–54), *n* = 1 study was a RCT (45), *n* = 4 studies were facility-based cross-sectional studies (40, 41, 43, 44), and *n* = 1 study was a (55) quasi-experimental time series.

Reports have targeted different types of interventions to improve the quality of MCH care. Based on the type of intervention, four studies focused on mobile and electronic health (eHealth) (38, 42, 45, 47), *n* = 5 studies focused on quality improvement standards (12, 38–40, 44), and *n* = 10 studies focused on human resource mobilization (training for healthcare providers, health extension workers, traditional birth attendants, and the community health development army, pregnant mothers, and supply material resources needed for MCH services) (43, 46, 48–55) (Table 1).

### Data extraction and synthesis intervention based on WHO's eight domains of quality care

Quality improvement interventions were summarized based on the WHO eight domains of quality care for mothers and newborns: most (15) interventions focused on domain 1 (evidence-based routine care and management of complications) (12, 38–41, 43–46, 50–55), 13 reports focused on domain 2 (the health information system enables the use of data to ensure early, appropriate action to improve the care of every woman and newborn) (38–41, 44–47, 51, 53–55), 9 studies focused on domain 7 (the availability of competent and motivated staff) (12, 38, 39, 45, 46, 52–54), 4 studies focused on domain 4 (effective communication) (38, 42, 46, 47), and 4 studies addressed the medicine and equipment required for maternal and newborn care (39, 43, 50, 52). Very few studies focusing on domains 3 (38), 5 (44, 48, 50), and 6 (50) were related to the functional referral system, promotion of respectful and dignified care, and emotional support, respectively (Table 2).

**Table 2:** Alignment of quality improvement intervention with WHO’s quality of maternal and newborn care standards.

Author(s) and year	Objective	WHO Framework for Quality of Maternal and Newborn Care (56)							
		Provision of care			Experience of acre			Both prevision and experience of care	
		Evidence-based practice for routine care and management of complication	Actionable information system	Functional referral system	Effective communication	Respect and preservation of dignity	Emotional support	Competent, motivated human resource	Availability of essential physical resources
Nigussie et al. (38)	To improve delivery, timeliness and coverage, quality, and referral of RMNCH services	✓	✓	✓	✓			✓	
	To bridge the communication gap between HCW and HEW using mHealth								
Hagaman et al. (12)	To evaluate the impact of QI health system interventions on MCH outcomes (feasibility of complex, low-cost, health-worker-driven improvement interventions)	✓						✓	
Ayalew et al. (39)	To see the effect of Standard-Based Management and Recognition (SBM-R) on MNH provider's performance	✓	✓					✓	✓
Biadgo et al. (40)	To assess the quality of maternal and neonatal healthcare providers using the national MCH quality care standards and strengthen and develop a sustainable, self-sufficient healthcare system	✓	✓						
Gebrehiwot and Tewolde (41)	To initiate a facility-based review of maternal deaths and near misses	✓	✓						
Kassa and Mokgadi (42)	To assess the effectiveness of the mHealth intervention in MCN quality care (improve communication between HCWs)				✓				
Dadi et al. (43)	To estimate the effect of place of ANC-1 visit and adherence to MOH's ANC visit recommendations, institutional delivery, and PNC	✓							✓
Getachew et al. (44)	To assess the care received by mothers and newborns during antenatal and delivery care	✓	✓			✓		✓	
Lund et al. (45)	To assess the effects of the safe delivery app (SDA) on perinatal survival and on healthcare workers' knowledge and skills in neonatal resuscitation	✓	✓					✓	
Sibley et al. (46)	To improve the completeness of maternal and newborn healthcare provided by the team of HEWs, community health development agents, and TBAs	✓	✓		✓			✓	
Desta et al., (47)	To see the effect of the mobile video show on community knowledge, attitudes, and beliefs toward MCH service utilization		✓		✓				
Asefa et al., (48)	To see the effectiveness of respectful maternity care (RMC) interventions					✓			
Mengistu et al. (49)									
Mihret et al. (50)	To reduce disrespectful and abusive maternal care	✓				✓	✓		✓
Berhanu et al. (51)	To assess the effect of CBNC on MCH services	✓	✓			✓			
Villadsen et al. (52)	To improve maternity care by ANC strengthening	✓						✓	✓
Tesfaye et al. (53)	To promote community maternal and newborn health (CMNH) family meetings and labor and birth notification to improve PNC	✓	✓		✓				
Lindtjørn et al. (54)	To assess the effects of several coordinated interventions (BEmOC and CEmOC) on effective coverage and reduce maternal deaths	✓	✓					✓	
Bitewulign et al. (55)	To evaluate the effect of integrating the use of the World Health Organization Safe Childbirth Checklist (WHO-SCC) into a district-wide system improvement collaborative program designed to improve and sustain the delivery of essential birth care practice	✓	✓					✓	
							
Total		15	13	1	6	3	1	9	4

## Discussion

Quality improvement of maternal and newborn care has been one of the national agenda in averting maternal and neonatal mortality. The current scoping review revealed that improving the quality of maternal and newborn care in Ethiopia is a complex and challenging task. Various sources have been identified, such as quality improvement strategies, ranging from community engagement to health system strengthening, including mHealth, community involvement, health education, standard-based practice, health workforce empowerment, and the supply of resources for MCH services. The key themes that emerged from the literature were the impact of mHealth interventions (safe delivery applications, SMS messages, and video shows), using MCH standards (WHO safe delivery checklist, quality standards, and reports), community involvement, and empowerment of healthcare providers for improving MCH care. However, a notable research gap exists regarding the impact of material resources, physical environment, and accessibility on the quality of maternal and newborn care, which needs further investigation.

The review studies suggested that the place of ANC-1 visit does not have a significant effect on the completion of the continuum of care; only 13.9% completed the continuum of care, with 6.6% of them receiving the Ministry of Health recommended ANC and only 25% attending PNC visits (7). This finding was supported by a study conducted in Ethiopia (43). Completion of the continuum of care may be influenced by factors other than the first ANC visit, such as socioeconomic status, mass media exposure, accessibility of the healthcare institution, and quality of care received during the first ANC visit (57, 58). Moreover, the flexibility of the healthcare system may influence women to seek their first ANC visit in alternative settings. Seeking care at a formal healthcare facility for an initial ANC visit can improve the likelihood of receiving timely and appropriate care throughout the continuum. Factors such as accurate risk assessment, early detection of complications, and effective referral systems are more likely to be present in formal healthcare settings (59). As such, it is essential to consider the holistic perspective encompassing multiple factors influencing the continuum of care.

The use of the Safe Childbirth Checklist (SCC) is associated with improved essential birth practice and reduced pregnancy complications, reducing the rate of severe pre-eclampsia (60, 61). According to a randomized control trial, the use of the WHO checklist had an impact on the safety culture among healthcare providers. The trial showed that healthcare providers were more likely to call attention to problems with patient care and report errors during periods of excessive workload when using the checklist (62). Evidence showed that knowledge and skills related to neonatal resuscitation deteriorate after 6 months of training (63), indicating the knowledge and skills of healthcare professionals should be emphasized greatly.

Community engagement and empowerment are also of paramount importance in improving quality care. Community involvement in decision-making processes and utilization related to MCH care helps ensure that services are responsive to their needs and preferences (64). Implementing community-oriented strategies improves skilled birth attendants (65), enhances knowledge and healthy behaviors related to MCH care (66), and reduces neonatal mortality (67). Improving maternal and child healthcare can be achieved by creating a peer support network. This approach can increase access to vital information, reduce isolation, and encourage positive health-seeking behavior (68). Community based outreach activities played a key role in identifying barriers to accessing care and improving MCH services (69). Emphasizing the involvement of the community is crucial for need assessment, community-led planning, and establishing a healthy community. Future efforts should prioritize community perspectives and involve them in culturally sensitive approaches.

The review identified technologies to improve maternal and newborn care, such as mHealth (using phone-based communication, SMS messaging, and mobile applications) to deliver healthcare services and information (38, 42, 45, 47). Studies also showed that SMS-based intervention could improve antenatal attendance, immunization rates, and mother's knowledge of MCH (70). SMS messages to pregnant women and new mothers can serve as reminders for assessing MCH services. Using mobile phone-based health behavior interventions in pregnancy improves behaviors, positive beliefs, and health outcomes (71, 72). Mobile health applications have proven beneficial for providers in making informed decisions while delivering care, collecting data, and providing health education (73, 74). Moreover, using voice counseling, job aid applications, direct messaging, and interactive media as a means of behavioral change communication had a significant impact on improving MCH care (75). Insufficient attention is given to intervention across different geographical areas. In addition, digital literacy, Internet, and electric sources must be addressed to ensure equitable access to mHealth. As such, focus should be given to the usability, applicability, and sustainability of mHealth for MCH services.

In addition, evidence showed that the quality of maternal and newborn care depends on facility readiness (infrastructure, supplies, health workforce, service delivery approach), adherence to guidelines, and provision of skilled care (76). However, the challenge lies in the equitable distribution of resources to ensure that all women, regardless of their geographical area, religion, or ethnicity, have access to quality maternity care. In summary, quality maternal and newborn care could be achieved through different partners’ involvement, prioritizing quality MCH services, promoting equity through universal healthcare coverage, improving facility capability, and strengthening the healthcare system through resources (5). Collecting, monitoring, and evaluating data are important for quality improvement in healthcare. Standardized indicators and metrics can help identify gaps, measure outcomes, and inform decision-making.

Despite the overall positive findings, it is important to note that most of the included studies focused on providing care (pre- and postinterventions). Moreover, the review focused on strategies for improving maternity care rather than assessing the effectiveness of quality interventions. The strength of this scoping review is the inclusion of both published and gray literature. The PRISMA-ScR checklist was used, with no restriction on the publication date. However, it is essential to acknowledge that language restriction was applied, which may introduce a potential bias. Moreover, literature was not searched from EMBASE, PsycINFO, CINHAL, HINARI, and Maternity and Infant Care databases due to their.

Future research should focus on the impact of the physical environment (healthcare setup, medical equipment, drugs, and supplies), culture, sustainability, and cost-effectiveness of interventions on the quality of MCH care. Long-term impact of quality intervention should also be investigated. In addition, the impact of healthcare providers’ knowledge, skills, attitudes, satisfaction, and adherence to MCH guidelines on quality maternal care should be considered. Projects focusing on capacity building, knowledge, and skill retention could significantly improve maternal and newborn care. Finally, mixed-method studies should be conducted to investigate the facilitation and barriers of quality improvement interventions for maternal and newborn care. Moreover, studies on emotional and functional referral systems to improve the quality of maternal and newborn care should be conducted.

## Conclusions

In conclusion, this scoping review identifies and maps various maternal and newborn quality improvement interventions in Ethiopia, focusing on mobile and electronic health, quality improvement standards, and human resource mobilization. This review found that community involvement, health education, mHealth, data-driven approaches, and strengthening the health system are crucial strategies for improving maternal and newborn care in Ethiopia. Future research should consider the impact of the physical environment, culture, sustainability, cost-effectiveness, and long-term effects of interventions, as well as healthcare providers’ knowledge, skills, attitudes, satisfaction, and adherence to guidelines.

## Data Availability

The original contributions presented in the study are included in the article/**Supplementary Material**; further inquiries can be directed to the corresponding author.
