# Supplementary material for: Interventions to improve the quality of maternal care in Ethiopia: a scoping review
Source: Front Glob Womens Health. 2024 Apr 17;5:1289835. doi: 10.3389/fgwh.2024.1289835 (PMC11061455; doi:10.3389/fgwh.2024.1289835)
Supplement: Supplementary file 2 [file Table2.docx]

Supplementary file 2: Search strategy

| Database | Search strategy | Result | Filter applied |
| --- | --- | --- | --- |
| Pub med | (Quality improvement intervention) or (QI project) or (quality indicator) or (mHealth intervention) or (provision of maternity care) or (client satisfaction) or (skilled maternity care) or (coverage) or (midwifery care) or safety or (compassionate and respectful maternity care) AND (maternal) or (pregnancy) or (antenatal) or (prenatal) or (intrapartum) or (delivery) or (postnatal) or (postpartum) AND (Ethiopia) | 6170 | Articles with English language |
