# Supplementary material for: Interventions to improve the quality of maternal care in Ethiopia: a scoping review
Source: Front Glob Womens Health. 2024 Apr 17;5:1289835. doi: 10.3389/fgwh.2024.1289835 (PMC11061455; doi:10.3389/fgwh.2024.1289835)
Supplement: Supplementary file 3 [file Table3.docx]

| **Author(s) & year** | **Study**  **settings** | Objective | **Study design** | **Intervention (s) type** | **Methods** | **Key findings** |
| --- | --- | --- | --- | --- | --- | --- |
| Nigussie et al. 2020 (1) | Amhara, SNNP, Oromia & Tigray | -Improve delivery, timeliness and coverage, quality, and referral of RMNCH services  -Bridged communication gap b/n HCW and HEW using mHealth | Process evaluation | Mobile and electronic health (mHealth) | - Provision of mobile for pregnant women, HEWs, and HCPs. - Providing training on the application - Performance monitoring and trace defaulter - mHealth applications-based supportive supervision - Registration and prioritization of maternity care services (ANC, delivery, and PNC services) - Providing automated job aids for HEWs - Referral and follow-up - mHealth information exchange between the health posts and health centers - Client’s notification of appointment reminders’ using SMS | - Improve real-time communication b/n health care providers. - Improve timely identification and registration of pregnant women. - Adherence to treatment protocols - Provides reliable, quality, and on-time data for action. - Supports access to clients’ previous and current clinical information. - Provides dynamic job aids to improve clinical skills and client counseling. |
| Hagaman AK et al, 2020 (2) | Amhara, SNNP, Oromia & Tigray | -To evaluate the impact of QI health systems intervention on MCH outcome (feasibility of complex, low-cost, health-worker-driven improvement  Interventions) | Quasi-experimental, interrupted time-series approach | Scaling QI health systems  Intervention  (From September 2016 - September  2018 (32 months)) | - QI initiative - Formation of quality improvement teams - QITs attended four structured learning sessions (Provide training on QI, Experience sharing, peer learning, and Intensive coaching followed by the implementation of team-initiated QI ‘change ideas and troubleshooting - data extraction from facility paper registers and validation | - Improved HCW’s adherence to a safe child practice - Increased attending at least four ANC visits from 64.1 to 75.3% - Increased rate of syphilis testing increased from 54.7 to 68.5% - Increase PNC visit within 48 h of discharge from 49.4 to 58.2% - Increase newborn-appropriate treatment in neonatal sepsis, kangaroo mother care, and birth asphyxia. - No impact on skilled delivery |
| Ayalew et al., 2017 (3) | Amhara, SNNP, Oromia & Tigray | To see the effect of Standard based management and recognition (SBM-R) on MNH providers performance | Post-only intervention and  comparison evaluation design | Standard-based management and recognition (SBM-R) quality improvement intervention  (From March 2011 to  June 2014) | - Introduce the SBM-R approach. - develop 10 technical areas and standards (80 standards for hospital and 79 for health center) - 3 round training on SBM-R for providers and managers - training on (BEmONC) and regular follow up - baseline assessment for gap identification - provide essential equipment’s and supply for MCH service. - establish quality improvement team - Regular follow up (Every 6 month) using direct structured observations, document review and provider interviews. - The ANC portion of the   tool consisted of 53 tasks in 8 skill areas; labor and delivery 105 tasks in 10 skill areas; and the PNC portion included 9 tasks in two skill areas | - Almost no difference in ANC performance (63.4% Vs 61.0%) - Average performance score for uncomplicated labor and delivery was higher among the intervention groups (77.5% versus 65.6%; p = 0.002) - Average performance score for immediate PNC service was higher among the intervention groups (72.8% versus 50.6%; p = 0.001) - Average performance score was higher among the intervention groups in rapid initial assessment (60.6% versus 42.8%, p = 0.019), care during labor (81.1% versus 66.0%, p = 0.001), and immediate newborn care (76.9% versus 61.9%, p = 0.013) |
| Biadgo et al., 2021 (4) | SNNP, Amhara, Oromia & Tigray | Assess the quality of maternal and neonatal health care provision using the national MCH quality care standards and strengthen and develop  sustainable, self-sufficient health care system | Facility based cross-sectional | Institute for healthcare improvement project using district wide collaborative approach | - Develop quality standards (28 items for input, 13 item for process, 4 items for outcome) related to health seeking behavior. - Establish quality health care collaborative demonstration and learning sites. - Identify gaps that affect performance. - Interview - Observation - Document review of patient cards | - The mean quality score of input (infrastructure, availability of equipment/supply, and essential drugs) was 62% - Quality of the process component was 43% - 53% of mothers were assessed for danger signs at admission. - Out of 1920 cases, only 38% of newborns were given vitamin K, and a mere 35% had skin-to-skin contact with their mothers and breastfed within one hour of delivery. - The quality of the maternal and neonatal health output component was 48% - 70% of births were attended by skilled health personnel. - Mean score for overall complication management was 38% - It was found that only 11% of postpartum hemorrhage cases were handled according to the protocol, and a significant 63% of females with pre-eclampsia received IV/IM MgSO4 treatment. - According to the established benchmark for quality of care in maternal and newborn health, a mere 15.6%, 9.3%, and 10.7% of healthcare facilities have successfully met the required standards for input, process, and output quality. - Hospitals and health centers achieved 79% and 59% of the input standards, 58% and 41% of the process standards, and 62% and 46% of the output standards, respectively. |
| Gebrehiwot Y et al., 2014 (5) | SNNP, Amhara, Oromia & Addis Ababa | To initiate facility-based review of maternal deaths and near misses | An in-depth review | Ethiopian Society of Obstetricians  and Gynecologists (ESOG) project of review maternal death  (Integrating the MDR and NMR process)  (November 1, 2010, and February 1, 2011,) | - Establish a technical review. - Standing committee - Maternal death and near miss review | - A total of 35047 deliveries, which included 7181 cesarean deliveries, 32,541 live births, and 2604 stillbirths. - A total of 2774 cases reviewed; 2568 were near misses and 206 maternal deaths. - The maternal mortality ratio (MMR) in the facilities was 728 per 100,000 live births. Additionally, the near-miss rate was 9079 per 100,000 live births and the case fatality rate was 8%. - 76% maternal deaths were attributed to direct obstetric causes and 7% due to indirect obstetric (4.8% were due to anemia) - 87.6% of women were critical ill and 4.4% women died on arrival, 70.2% women were delayed at home, 48.1% delayed due to lack of transportation to reach the nearest appropriate health facility, and 34.7% delay in receiving care owing to a shortage of skilled health professionals or a lack of appropriate medical supplies. - 23.6% women who sought help and medical attention had no access to appropriate health facilities. - 55.9% women did not receive prenatal care. - Date of delivery was registered for 1434 (51.7%) women, while date of discharge was recorded for 1356 (48.9%) - The ratio of live births to stillbirths among the delivered cases (n=2124) was 1:2 - The partograph was used for only 219 (39.9%) eligible cases (n = 549) |
| Kassa A et al.,2022 (6) | North wollo, Amhara region | To assess effectiveness of the mHealth intervention in MCN quality care  (Improve  communication between HCWs) | Pre-post intervention | mHealth  for 24 months | - Web application for register and SMS engine for HCWs - Visiting pregnant women at Health Center marked with unique identification number. - The system generates four SMS reminders in connection with ANC visit based on calculation of GA (at GA of 26, 32,36, and 39 weeks); one SMS at 30, 14, 6, and 2 weeks; at day 1, day 3, and day 7 of PNC; three schedules at 6th, 10th, and 14th weeks for Penta vaccine | - Improved in 4 and above ANC visit (13.8% at baseline to 64% after mHealth intervention) - Timeliness to start (44.5% and 77.3%) - Institutional delivery (35.0% and 71.2% - PNC within 6 hours of birth (23.8% at baseline and 84%) - Penta-3 vaccination coverage (61.5% & 70.4%) |
| Dadi et al., 2021(7) | In 9 regions of Ethiopia | To estimate effect of place of ANC-1 visit and adherence to MOH’s ANC visit recommendations, institutional delivery and PNC. | National HEP assessment survey (secondary data) | Health extension program | Data collected during health extension program | - Place of ANC-1 visit does not have a significant effect on the completion of continuum of care. - The mean availability score of medical equipment at health post was 7.98 and 1.44% of the HPs do not have any medical equipment’s. - Women who have at least ANC-1, 14.8% completed CoC. - More than half, 55.5% the women were not told at least one danger sign on their previous pregnancy. - Two third (64%) of women delivered their second youngest child at home. - 92% took at least one ANC visit, but only 25% took PNC. - Only 13.88% of the cohort completed the continuum of   care, 6.6% of them received MOH recommended ANC.   - Adherence of ANC visit to the MOH recommendations improve continuum of care |
| Getachew et al., 2-11 (8) | In 9 regions of Ethiopia | To assess the care received by mothers and newborns during antenatal and delivery care | Institution based survey | Standard care | - Interview and observation | - About 29% of women received the full components of AMTSL - Low knowledge of PPH management - Magnesium sulfate was rarely available in labor and delivery wards (only available on 3 facility/ out of 19 facilities - Partograph use was very low. - Knowledge of the signs of obstructed labor was low. - diagnoses and management of asphyxia was low. - Providers asked the client about at least one danger sign in only 34% of the cases observed. - Providers asked the client about at least one complication during previous pregnancies in 27% of cases. - Only half of the women in labor were greeted respectfully by the provider, and only 13% of women were asked by the provider if they had any questions. - 66% of women were supported by the provider during labor in a friendly manner. - Explanations of procedures and what would happen during labor were offered in about 35% of the labors observed. - Only 44% of the women were draped to protect their privacy. - Only 12% of newborns were placed skin-to-skin. - Only 18% of newborns received all elements of essential newborn care. - only 48% adherence to thermal control |
| Lund S et al. , 2016 (9) | Oromia region | effects of the safe delivery app (SDA) on perinatal survival and  on health care workers’ knowledge and skills in neonatal resuscitation | Randomized control trial | SDA mHealth training | - Mobile phone intervention with safe delivery application (SDA) - Standard care - Training on basic emergency obstetric and neonatal care - Data collection for secondary outcomes 6 and 12 months - Follow women from delivery to 7 for perinatal outcome | - SDA was an effective method to improve and sustain the health care workers’ knowledge and skills in neonatal resuscitation. - Perinatal mortality was not significantly reduced after the intervention |
| Sibley LM et al., 2014 (10) | Amhara and Oromia | To improve completeness of maternal and newborn health care provided by the team of HEWs, community health development agents, and TBAs | Project evaluation (pre-post intervention) | Maternal Health in Ethiopia Partnership (MaNHEP)project  (3.5 years project) | - Community based maternal and newborn health training program, continuous quality improvement, behavior change communications. - Monthly quality improvement monitoring | - Improvements in the completeness of maternal and newborn health care provision - Improved providers confidence and skill of birth care - Improved identification of pregnant women, enrollment of pregnant women in ANC - Improved perinatal outcomes |
| Desta et al., 2014 (11) | Amhara and Oromia | To see the effect of the mobile video  show on community knowledge, attitudes, and beliefs towards MCH service utilization | Project evaluation  (Qualitative and quantitative) | MaNHEP project | - Use mobile video show for behavioral change on MCH | - Mobile Video show promotes access to behavior change communication on MCH, bringing about desired changes in knowledge and beliefs. - Improve recalling of maternal complications - Retain Key messages |
| Asefa A et al., 2020 (12)  Mengistu B et al., 2021 (13) | SNNPR, Tigray & Oromia | To see the effectiveness of Respectful maternity care (RMC) interventions | Interventional mixed methods design (Pre post quantitative and post intervention qualitative) | Respectful maternity care project | - Training manual development and RMC videos - 3 days training on respectful maternity care - Consultative meeting with managers - Coaching | - Prior to the intervention, it was reported that 39.1% of participants witnessed examinations without privacy and 21.9% reported the use of physical force. Additionally, 29.7% admitted women mistreated. - The awareness of women's rights during childbirth and their perceptions and attitudes towards RMC have been improved among providers. - Positive perception on 8 RMC domains increased from 21.9% to 35.9% - Belief on not necessary to seek verbal consent from a woman prior to conducting a vaginal examination (15.6% pre- test Vs 10.9% during posttest) - It was believed that nurses and doctors were unable to alter the procedures in the delivery room, and this perception worsened from 17.2% in the pre-test to 18.7% in the post-test. - The video helped providers to see their care from their clients’ perspectives. - The training helped on potential root causes of mistreatment and develop solutions. |
| Mihret H et al., 2020 (14) | Amhara | Reducing disrespectful and abusive maternal care | Pre-post intervention mixed method | RMC Project | - Route cause analysis at baseline - Provision of 5-day training on RMC - Prepare of standard written guidelines and protocols on RMC - Waiting room construction - Improving infrastructure such as; availing screening or curtain, equipment, essential drugs and supplies - Supportive supervision and mentoring, and staff motivation | - Initially, there are inadequate monitoring and evaluation systems, insufficient knowledge, and skills among staff regarding respectful care, and low provider motivation. Additionally, there are missing medical equipment such as ultrasound and blood pressure apparatus, delivery coaches, and crucial drugs supplies needed for maternal health services, and poor working environment. - In addition, there is a lack of written policies detailing the responsibilities of healthcare providers in the RMC process, as well as a professional code of conduct and ethics for providers working in labour wards and ANC clinics. Furthermore, there is an inadequate system in place for reporting illegal, incompetent, or impaired practices. - Disrespect and abuse during pregnancy and childbirth decreased by 55.9% (reducing from 71.8% to 15.9%) - Physical abuse during maternal care was reduced from 61% to 15.4% - Non-confidentiality care, discrimination care, and abandonment or denial of care reduced from the baseline by 54.8%, 59.3% and 68.4%, respectively. - Non-consented care domain decreased by 54.9% |
| Berhanu D et al., 2021 (15) | SNNP, Amhara, Oromia & Tigray | Effect of CBNC on MCH services | Program evaluation (  pre-post survey) | Community-Based Newborn Care (CBNC) programme | - Training of HEWs on 9 components of CBNC - Reporting high utilization for integrated CBNC - Having a strong linkage within their primary health care units - Having a well-established health extension programme - Having functional Women’s Development Army networks. | - The percentage of women who had at least one ANC visit increased by 15%, those who had four or more visits increased by 17% (from 36% to 53%), and 40% increase in the promotion of institutional delivery (from 22% to 62%). - Among women with at least one ANC, the proportion who reported giving a urine sample increased by 18%, receiving a syphilis test by 8%, and receiving iron folate by 9%. - There has been a decline in the percentage of women who reported receiving guidance on birth preparedness by 7%, nutrition advice by 10%, and HIV testing by 19%. - The percentage of newborns receiving PNC visits within 48 hours of birth has decreased. There has been a 6% decrease for home deliveries and a 14% decrease for institutional deliveries. - Skin to skin contact increased by 11 % - Delay newborn’s bathing increased by 14% for home delivery |
| Villadsen SF et al, 2015 (16) | Oromia | ANC strengthen to improve maternity care | Pre and post intervention  (ANC intervention) |  | - Supply equipment needed for ANC - Trainings of health staff and laboratory staffs - Development of health education materials - Seminar with TBAs - Adaption of guideline - Supervision | - Improved health education on danger signs during pregnancy - Improved laboratory testing (urine test and blood tests other than HIV) - Improved health problem identification - Increased in the proportion of women waiting less than one hour. - Improved women satisfaction with the service - No effect on the frequency of physical examination performed and conduct of health professionals |
| Tesfaye S et al., 2014 (17) | Amhara & Oromia | Promotion of community maternal and newborn health  (CMNH) family meetings and labor and birth notification contributed to improve PNC | baseline and end-line cross-sectional survey | A Community-based, Collaborative Quality Improvement  Approach (MaNHEP) project | - Training HEWs, community health development agents, and traditional birth attendants (TBAs) in maternal care - Training for pregnant women and their family care giver in their 2^nd^ and 3^rd^ trimesters in maternal and newborn care including PNC through community maternal and newborn health (CMNH) family meetings - Collaborative quality improvement focusing on promotion of pregnancy identification, antenatal care registration, CMNH family meeting attendance, labor and birth notification and PNC within 48 hours of birth by a HEWs. - Behavior change communications | - The percentage of newborns receiving a PNC from skilled providers or HEWs within 48 hours of birth significantly increased in Amhara (from 5% to 51%) and Oromiya (from 15% to 47%). - Women who received any ANC from a skilled provider or HEWs more likely to receive PNC - Women who participated in two or more CMNH family meetings alongside their family members had a significantly higher chance of receiving PNC within 48 hours from a skilled provider compared to those who attended less than two meetings. Women whose most recent birth was attended by HEWs/HCWs receive PNC |
| Lindtjørn B et al, 2017 (18) | SNNPR | Effects of several coordinated interventions (BEmOC and CEmOC) on effective coverage and reduce  maternal deaths. | Pre post intervention | health-care system strengthening  interventions | - Equipping institutions with trained staff on BEmOC and CEmOC, provide essential and basic equipment. - Regular monitoring and supervision - community-based birth registration system | - During the intervention period, there was a significant decrease in MMR, with a decline of 64% from 477 to 219 deaths per 100,000 live births. The reduction in MMR was particularly pronounced in woreda where CEmOC functions. - Four or more antenatal controls increased by 20% - There has been an improvement in the number of women referred for delivery service. The percentage of women delivering at home decreased by 20.4%, from 89.8% to 69.2%. - Decline in the use of traditional birth attendants. - Number of women referred to hospitals increased by 3.3%, and to health centers by 7.2% more in CEmOC areas. - Reduce stillbirths by 46% (from 14.5 to 7.8 per 1000 births) - Those having road access to health facility near to health facility had lower mortality risk |
| Bitewulign B et al, 2021 (19) | Amhara, Oromia, Tigray & SNNP | Evaluates the effect of integrating the use of the World Health Organization Safe Childbirth Checklist  (WHO-SCC) into a district-wide system improvement collaborative program designed to improve and sustain the  delivery of essential birth care practice | Time series study |  | - Integrating the use of the World Health Organization Safe Childbirth Checklist (WHO-SCC) - Training on the checklist - Three “clinical bundles” were created from the WHO-SCC: On admission, before pushing, and soon after Birth bundles - Assess adherence monthly by reviewing charts of live births. - Coaching - observation - Document review - Triangulation of the checklist with the document review | - Improved adherence and quality of labor and delivery |

References

1. Nigussie ZY, Zemicheal NF, Tiruneh GT, Bayou YT, Teklu GA, Kibret ES, et al. Using mHealth to Improve Timeliness and Quality of Maternal and Newborn Health in the Primary Health Care System in Ethiopia. Global Health: Science and Practice. 2021;9(3):668-81.

2. Hagaman AK, Singh K, Abate M, Alemu H, Kefale AB, Bitewulign B, et al. The impacts of quality improvement on maternal and newborn health: preliminary findings from a health system integrated intervention in four Ethiopian regions. BMC health services research. 2020;20(1):1-12.

3. Ayalew F, Eyassu G, Seyoum N, van Roosmalen J, Bazant E, Kim YM, et al. Using a quality improvement model to enhance providers’ performance in maternal and newborn health care: a post-only intervention and comparison design. BMC pregnancy and childbirth. 2017;17(1):1-9.

4. Biadgo A, Legesse A, Estifanos AS, Singh K, Mulissa Z, Kiflie A, et al. Quality of maternal and newborn health care in Ethiopia: a cross-sectional study. BMC health services research. 2021;21(1):1-10.

5. Gebrehiwot Y, Tewolde BT. Improving maternity care in Ethiopia through facility based review of maternal deaths and near misses. International Journal of Gynecology & Obstetrics. 2014;127:S29-S34.

6. Kassa A, Mokgadi M. Effectiveness of mHEALTH Application at Primary Health Care to Improve Maternal and New-born Health Services in Rural Ethiopia: Comparative study. medRxiv. 2022.

7. Dadi TL, Medhin G, Kasaye HK, Kassie GM, Jebena MG, Gobezie WA, et al. Continuum of maternity care among rural women in Ethiopia: does place and frequency of antenatal care visit matter? Reproductive health. 2021;18(1):1-12.

8. Getachew A, Ricca J, Cantor D, Rawlins B, Rosen H, Tekleberhan A, et al. Quality of care for prevention and management of common maternal and newborn complications: a study of Ethiopia’s hospitals. Baltimore: Jhpiego. 2011;6:1-9.

9. Lund S, Boas IM, Bedesa T, Fekede W, Nielsen HS, Sørensen BL. Association between the safe delivery app and quality of care and perinatal survival in Ethiopia: a randomized clinical trial. JAMA pediatrics. 2016;170(8):765-71.

10. Sibley LM, Tesfaye S, Fekadu Desta B, Hailemichael Frew A, Kebede A, Mohammed H, et al. Improving maternal and newborn health care delivery in rural amhara and oromiya regions of ethiopia through the maternal and newborn health in ethiopia partnership. Journal of Midwifery & Women's Health. 2014;59(s1):S6-S20.

11. Desta BF, Mohammed H, Barry D, Frew AH, Hepburn K, Claypoole C. Use of mobile video show for community behavior change on maternal and newborn health in rural Ethiopia. Journal of Midwifery & Women's Health. 2014;59(s1):S65-S72.

12. Asefa A, Morgan A, Bohren MA, Kermode M. Lessons learned through respectful maternity care training and its implementation in Ethiopia: an interventional mixed methods study. Reproductive health. 2020;17(1):1-12.

13. Mengistu B, Alemu H, Kassa M, Zelalem M, Abate M, Bitewulign B, et al. An innovative intervention to improve respectful maternity care in three Districts in Ethiopia. BMC pregnancy and childbirth. 2021;21(1):1-10.

14. Mihret H, Atnafu A, Gebremedhin T, Dellie E. Reducing disrespect and abuse of women during antenatal care and delivery services at injibara general hospital, Northwest Ethiopia: a pre–post interventional study. International Journal of Women's Health. 2020;12:835.

15. Berhanu D, Allen E, Beaumont E, Tomlin K, Taddesse N, Dinsa G, et al. Coverage of antenatal, intrapartum, and newborn care in 104 districts of Ethiopia: A before and after study four years after the launch of the national Community-Based Newborn Care programme. Plos one. 2021;16(8):e0251706.

16. Villadsen SF, Negussie D, GebreMariam A, Tilahun A, Friis H, Rasch V. Antenatal care strengthening for improved quality of care in Jimma, Ethiopia: an effectiveness study. BMC Public Health. 2015;15(1):1-13.

17. Tesfaye S, Barry D, Gobezayehu AG, Frew AH, Stover KE, Tessema H, et al. Improving coverage of postnatal care in rural Ethiopia using a community-based, collaborative quality improvement approach. J Midwifery Womens Health. 2014;59(1):12168.

18. Lindtjørn B, Mitiku D, Zidda Z, Yaya Y. Reducing maternal deaths in Ethiopia: results of an intervention Programme in Southwest Ethiopia. PLoS One. 2017;12(1):e0169304.

19. Bitewulign B, Abdissa D, Mulissa Z, Kiflie A, Abate M, Biadgo A, et al. Using the WHO safe childbirth checklist to improve essential care delivery as part of the district-wide maternal and newborn health quality improvement initiative, a time series study. BMC Health Services Research. 2021;21(1):1-11.
