# Supplementary material for: Interventions to improve the quality of maternal care in Ethiopia: a scoping review
Source: Front Glob Womens Health. 2024 Apr 17;5:1289835. doi: 10.3389/fgwh.2024.1289835 (PMC11061455; doi:10.3389/fgwh.2024.1289835)
Supplement: Supplementary file 4 [file Table4.docx]

| Author(s) & year | Objective | WHO Framework for Quality of Maternal and Newborn Care ([20](#_ENREF_20)) | | | | | | | |
| --- | --- | --- | --- | --- | --- | --- | --- | --- | --- |
|  |  | **Provision of care** | | | **Experience of acre** | | | **Both prevision and experience of care** | |
|  |  | **Evidence-based practice for routine care and management of complication** | **Actionable information System** | **Functional referral system** | **Effective communication** | **Respect and preservation of dignity** | **Emotional support** | **Competent, motivated human resource** | **Essential physical resources available** |
| Nigussie et al. 2020 (1) | Improve delivery, timeliness and coverage, quality, and referral of RMNCH services.  Bridged communication gap b/n HCW and HEW using mHealth |  |  |  |  |  |  |  |  |
| Hagaman AK et al, 2020 (2) | To evaluate the impact of QI health systems intervention on MCH outcome (feasibility of complex, low-cost, health-worker-driven improvement Interventions) |  |  |  |  |  |  |  |  |
| Ayalew et al., 2017 (3) | To see the effect of Standard based management and recognition (SBM-R) on MNH provider’s performance |  |  |  |  |  |  |  |  |
| Biadgo et al., 2021 (4) | Assess the quality of maternal and neonatal health care providers using the national MCH quality care standards and strengthen and develop a sustainable, self-sufficient health care system. |  |  |  |  |  |  |  |  |
| Gebrehiwot Y et al., 2014 (5) | To initiate a facility-based review of maternal deaths and near misses |  |  |  |  |  |  |  |  |
| Kassa A et al.,2022 (6) | To assess the effectiveness of the mHealth intervention in MCN quality care  (Improve communication between HCWs) |  |  |  |  |  |  |  |  |
| Dadi et al., 2021(7) | To estimate effect of place of ANC-1 visit and adherence to MOH’s ANC visit recommendations, institutional delivery, and PNC |  |  |  |  |  |  |  |  |
| Getachew et al., 2-11 (8) | To assess the care received by mothers and newborns during antenatal and delivery care |  |  |  |  |  |  |  |  |
| Lund S et al. , 2016 (9) | effects of the safe delivery app (SDA) on perinatal survival and on health care workers’ knowledge and skills in neonatal resuscitation |  |  |  |  |  |  |  |  |
| Sibley LM et al., 2014 (10) | To improve completeness of maternal and newborn health care provided by the team of HEWs, community health development agents, and TBAs |  |  |  |  |  |  |  |  |
| Desta et al., 2014 (11) | To see the effect of the mobile video  show on community knowledge, attitudes, and beliefs towards MCH service utilization |  |  |  |  |  |  |  |  |
| Asefa A et al., 2020 (12)  Mengistu B et al., 2021 (13) | To see the effectiveness of Respectful maternity care (RMC) interventions |  |  |  |  |  |  |  |  |
| Mihret H et al., 2020 (14) | Reducing disrespectful and abusive maternal care |  |  |  |  |  |  |  |  |
| Berhanu D et al., 2021 (15) | Effect of CBNC on MCH services |  |  |  |  |  |  |  |  |
| Villadsen SF et al, 2015 (16) | ANC strengthen to improve maternity care |  |  |  |  |  |  |  |  |
| Tesfaye S et al., 2014 (17) | Promotion of community maternal and newborn health (CMNH) family meetings and labor and birth notification contributed to improve PNC |  |  |  |  |  |  |  |  |
| Lindtjørn B et al, 2017 (18) | Effects of several coordinated interventions (BEmOC and CEmOC) on effective coverage and reduce maternal deaths. |  |  |  |  |  |  |  |  |
| Bitewulign B et al, 2021 (19) | Evaluates the effect of integrating the use of the World Health Organization Safe Childbirth Checklist  (WHO-SCC) into a district-wide system improvement collaborative program designed to improve and sustain the  delivery of essential birth care practice |  |  |  |  |  |  |  |  |
| Total |  | 15 | 13 | 1 | 6 | 3 | 1 | 9 | 4 |

1. Nigussie ZY, Zemicheal NF, Tiruneh GT, Bayou YT, Teklu GA, Kibret ES, et al. Using mHealth to Improve Timeliness and Quality of Maternal and Newborn Health in the Primary Health Care System in Ethiopia. Global Health: Science and Practice. 2021;9(3):668-81.

2. Hagaman AK, Singh K, Abate M, Alemu H, Kefale AB, Bitewulign B, et al. The impacts of quality improvement on maternal and newborn health: preliminary findings from a health system integrated intervention in four Ethiopian regions. BMC health services research. 2020;20(1):1-12.

3. Ayalew F, Eyassu G, Seyoum N, van Roosmalen J, Bazant E, Kim YM, et al. Using a quality improvement model to enhance providers’ performance in maternal and newborn health care: a post-only intervention and comparison design. BMC pregnancy and childbirth. 2017;17(1):1-9.

4. Biadgo A, Legesse A, Estifanos AS, Singh K, Mulissa Z, Kiflie A, et al. Quality of maternal and newborn health care in Ethiopia: a cross-sectional study. BMC health services research. 2021;21(1):1-10.

5. Gebrehiwot Y, Tewolde BT. Improving maternity care in Ethiopia through facility based review of maternal deaths and near misses. International Journal of Gynecology & Obstetrics. 2014;127:S29-S34.

6. Kassa A, Mokgadi M. Effectiveness of mHEALTH Application at Primary Health Care to Improve Maternal and New-born Health Services in Rural Ethiopia: Comparative study. medRxiv. 2022.

7. Dadi TL, Medhin G, Kasaye HK, Kassie GM, Jebena MG, Gobezie WA, et al. Continuum of maternity care among rural women in Ethiopia: does place and frequency of antenatal care visit matter? Reproductive health. 2021;18(1):1-12.

8. Getachew A, Ricca J, Cantor D, Rawlins B, Rosen H, Tekleberhan A, et al. Quality of care for prevention and management of common maternal and newborn complications: a study of Ethiopia’s hospitals. Baltimore: Jhpiego. 2011;6:1-9.

9. Lund S, Boas IM, Bedesa T, Fekede W, Nielsen HS, Sørensen BL. Association between the safe delivery app and quality of care and perinatal survival in Ethiopia: a randomized clinical trial. JAMA pediatrics. 2016;170(8):765-71.

10. Sibley LM, Tesfaye S, Fekadu Desta B, Hailemichael Frew A, Kebede A, Mohammed H, et al. Improving maternal and newborn health care delivery in rural amhara and oromiya regions of ethiopia through the maternal and newborn health in ethiopia partnership. Journal of Midwifery & Women's Health. 2014;59(s1):S6-S20.

11. Desta BF, Mohammed H, Barry D, Frew AH, Hepburn K, Claypoole C. Use of mobile video show for community behavior change on maternal and newborn health in rural Ethiopia. Journal of Midwifery & Women's Health. 2014;59(s1):S65-S72.

12. Asefa A, Morgan A, Bohren MA, Kermode M. Lessons learned through respectful maternity care training and its implementation in Ethiopia: an interventional mixed methods study. Reproductive health. 2020;17(1):1-12.

13. Mengistu B, Alemu H, Kassa M, Zelalem M, Abate M, Bitewulign B, et al. An innovative intervention to improve respectful maternity care in three Districts in Ethiopia. BMC pregnancy and childbirth. 2021;21(1):1-10.

14. Mihret H, Atnafu A, Gebremedhin T, Dellie E. Reducing disrespect and abuse of women during antenatal care and delivery services at injibara general hospital, Northwest Ethiopia: a pre–post interventional study. International Journal of Women's Health. 2020;12:835.

15. Berhanu D, Allen E, Beaumont E, Tomlin K, Taddesse N, Dinsa G, et al. Coverage of antenatal, intrapartum, and newborn care in 104 districts of Ethiopia: A before and after study four years after the launch of the national Community-Based Newborn Care programme. Plos one. 2021;16(8):e0251706.

16. Villadsen SF, Negussie D, GebreMariam A, Tilahun A, Friis H, Rasch V. Antenatal care strengthening for improved quality of care in Jimma, Ethiopia: an effectiveness study. BMC Public Health. 2015;15(1):1-13.

17. Tesfaye S, Barry D, Gobezayehu AG, Frew AH, Stover KE, Tessema H, et al. Improving coverage of postnatal care in rural Ethiopia using a community-based, collaborative quality improvement approach. J Midwifery Womens Health. 2014;59(1):12168.

18. Lindtjørn B, Mitiku D, Zidda Z, Yaya Y. Reducing maternal deaths in Ethiopia: results of an intervention Programme in Southwest Ethiopia. PLoS One. 2017;12(1):e0169304.

19. Bitewulign B, Abdissa D, Mulissa Z, Kiflie A, Abate M, Biadgo A, et al. Using the WHO safe childbirth checklist to improve essential care delivery as part of the district-wide maternal and newborn health quality improvement initiative, a time series study. BMC Health Services Research. 2021;21(1):1-11.
